# Chagas Cardiomyopathy Manifestations and *Trypanosoma cruzi* Genotypes Circulating in Chronic Chagasic Patients

**DOI:** 10.1371/journal.pntd.0000899

**Published:** 2010-11-30

**Authors:** Juan David Ramírez, Felipe Guhl, Lina María Rendón, Fernando Rosas, Jose A. Marin-Neto, Carlos A. Morillo

**Affiliations:** 1 Centro de investigaciones en Microbiología y Parasitología Tropical (CIMPAT), Facultad de Ciencias, Departamento de Ciencias Biológicas, Universidad de los Andes, Bogotá, Colombia; 2 Electrofisiología, Clínica Abood Shaio, Bogotá, Colombia; 3 Cardiology Division, Internal Medicine Department, Medical School of Ribeirao Preto, Universidad de Sao Paulo, Sao Paulo, Brazil; 4 Department of Medicine, Cardiology Division, McMaster University, PHRI-HHSC, Hamilton, Ontario, Canada; National Institutes of Health, United States of America

## Abstract

Chagas disease caused by *Trypanosoma cruzi* is a complex disease that is endemic and an important problem in public health in Latin America. The *T. cruzi* parasite is classified into six discrete taxonomic units (DTUs) based on the recently proposed nomenclature (TcI, TcII, TcIII, TcIV, TcV and TcVI). The discovery of genetic variability within TcI showed the presence of five genotypes (Ia, Ib, Ic, Id and Ie) related to the transmission cycle of Chagas disease. In Colombia, TcI is more prevalent but TcII has also been reported, as has mixed infection by both TcI and TcII in the same Chagasic patient. The objectives of this study were to determine the *T. cruzi* DTUs that are circulating in Colombian chronic Chagasic patients and to obtain more information about the molecular epidemiology of Chagas disease in Colombia. We also assessed the presence of electrocardiographic, radiologic and echocardiographic abnormalities with the purpose of correlating *T. cruzi* genetic variability and cardiac disease. Molecular characterization was performed in Colombian adult chronic Chagasic patients based on the intergenic region of the mini-exon gene, the 24Sα and 18S regions of rDNA and the variable region of satellite DNA, whereby the presence of *T.cruzi* I, II, III and IV was detected. In our population, mixed infections also occurred, with TcI-TcII, TcI-TcIII and TcI-TcIV, as well as the existence of the TcI genotypes showing the presence of genotypes Ia and Id. Patients infected with TcI demonstrated a higher prevalence of cardiac alterations than those infected with TcII. These results corroborate the predominance of TcI in Colombia and show the first report of TcIII and TcIV in Colombian Chagasic patients. Findings also indicate that Chagas cardiomyopathy manifestations are more correlated with TcI than with TcII in Colombia.

## Author Summary

*Trypanosoma cruzi* the aetiological agent of Chagas disease infects over 8 million people in Latin America. Currently, six genetic groups or DTUs have been identified in this highly genetic and diverse parasite. Many authors have considered that disease installation is induced by this genetic variability in *T. cruzi* but few comparisons have been made to make an approach of this premise. We performed an analysis including 240 chronic ascertained Chagasic patients evaluating cardiac alterations in electrocardiogram, radiology and echocardiogram. Also, we developed molecular characterisation on samples from these patients showing that in Colombia *T.cruzi* I is the predominant but others such as TcII, TcIII and TcIV can be found in low proportions as mixed infections TcI/TcII-TcIV. We conclude that TcI is more related to cardiomyopathy than TcII and we show the first report of TcIII and IV in chronic Chagasic patients from Colombia. These results will help to elucidate the molecular epidemiology of *T. cruzi* in this country.

## Introduction

Chagas disease caused by the parasite *Trypanosoma cruzi* is a complex zoonosis that is widely distributed throughout the American continent. The infection can be acquired by triatomine faeces, blood transfusion, oral and congenital transmission and by laboratory accidents. Chagas disease represents an important public health problem, with estimates by the Pan American Health Organization in 2005 of at least 7.7 million people having *T. cruzi* infection and other 110 million being at risk [1]. Also, immigration of infected people from endemic countries is now making Chagas disease a relevant health issue in other regions, including Europe and the United States [2]. Chagas disease comprises two stages where the acute phase occurs about one week after initial infection and about 30–40% of the infected patients develop the chronic phase of the disease, when the cardiomyopathy is the most frequent and severe clinical manifestation. [2]

The *T. cruzi* parasite comprises a heterogeneous population that displays clonal propagation due to the different cycles of transmission, and the possibility of recombination exchange that can be found in nature and has been previously reported *in vitro* [3], [4], [5]. Recently a new nomenclature for *T. cruzi* has been adopted and includes six Discrete Taxonomic Units (DTUs) named as *T. cruzi* I (TcI), *T. cruzi* II (TcII), *T. cruzi* III (TcIII), *T. cruzi* IV (TcIV), *T. cruzi* V (TcV) and *T. cruzi* VI (TcVI) based on different molecular markers and biological features [6]. Recent studies based on mini-exon gene sequences have shown polymorphism on this region reporting four genotypes within TcI Colombian isolates, these genotypes have also been reported in other regions of South America where five TcI genotypes have been detected [7]. Also different molecular markers including a 48 set of microsatellite loci have shown the great diversity in TcI [8], [9], [10], [11]. Primers designed based on the sequences of TcI Colombian isolates confirmed the existence of three genotypes (Ia, Ib and Id) and a new genotype found in the southern cone countries named as TcIe [7], [12], also the use of Internal Transcribed Spacers 1 and 2 clustered the genotypes Ia, Ib and Id as being related to transmission cycles of Chagas disease [13]. Genetic variability has been clearly demonstrated in *T. cruzi* reporting homogeneous (TcII) and heterogeneous groups considered hybrids due to recombination events (TcIII-TcVI) [3], [4], [5], [14], [15], [16], [17]. Hybrids are considered within *T. cruzi* showing TcV and TcVI as products of recombination of TcII and TcIII and TcIII/TcIV as potential products of recombination of TcI and TcII [5], [16], although this last statement is still controversial.

The molecular epidemiology of *T. cruzi* may have important implications on the disease features. However, few correlations have been relating *T. cruzi* genetic variability and the disease outcome, showing TcI more related to patients with cardiomyopathy in Colombia and Venezuela and TcII-TcVI more related to patients with digestive syndrome (megaesophagus/megacolon) [2], [18]. In Colombia, TcI is predominant in patients, insect vectors and reservoirs, but TcII has also been reported. The first description of nine chronic Chagasic patients infected with TcII was reported by Zafra *et al*., 2008 [19] and also mixed infection with TcI and TcII in the same patient was reported [20]. The direct detection of *T. cruzi* DTUs in the blood of chronic Chagasic patients was established by amplification of the 24Sα rDNA divergent domain and the use of mitochondrial house-keeping genes [19]. In this study, molecular characterisation of *T. cruzi* DTUs showed that most of the patients were infected with TcI and some patients were found infected with TcII (9.9%). Recently, a new approach of *T. cruzi* DTUs detection in chronic Chagasic patients was developed showing that TcI was the predominant DTU and TcII was also detected reporting that the genetic characteristics of the TcII parasites found in Colombia were similar to those TcII found in Bolivia and Chile [21].

The objective of our study was to characterise and determine *T. cruzi* DTUs in chronic Chagasic patients from Colombia and to correlate the molecular variability of the parasite with the presence or absence of cardiac disease manifestations exhibited by the patients.

## Methods

### Ethics statement, sample collection and DNA isolation

A total of 240 seropositive chronic Chagasic patients were included in the study, as part of the Colombian population recruited for the BENEFIT trial (BENznidazol Evaluation For Interrupting Trypanosomiasis project (BENEFIT). Samples were taken as part of the main BENEFIT trial that has recruited to date 2150 patients from Argentina, Bolivia, Brazil, El Salvador and Colombia. Written and oral consent was obtained in all patients included as part of the BENEFIT trial, the study is approved by all local and national IRBs. Furthermore the study is approved by the Ethics Research Committe of the WHO as one of the funding agencies of the BENEFIT trial, [22]. All patients regardless of a positive or negative baseline PCR are being followed as part of the main trial which outcome is a composite of clinical events referenced in the text [22]. Following the inclusion and exclusion criteria for the BENEFIT study, all patients had cardiomyopathy, as defined by the pre-established ECG or Echo abnormalities. Twenty serologically negative control patients from non-endemic regions were also included. A 10-mL blood sample was collected from all patients and control subjects. Blood samples were mixed with an equal volume of 6 M guanidine HCl/0.2 M EDTA solution immediately after sample collection. The samples were immersed in boiling water for 15 min. After cooling, two 200-µL aliquots were taken from each patient blood lysate and successive phenol-chloroform extractions were performed on this material as previously reported [23]. The DNA was then stored at −20°C. The DNA purity and concentrations were determined using an Eppendorf Biophotometer 6131 at 260/280 nm.

### *T. cruzi* molecular detection and molecular characterisation

**Figure 1:**
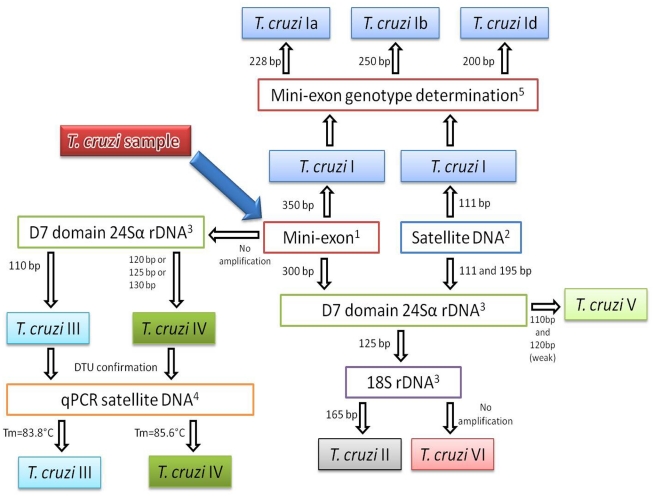
Algorithm for molecular characterization of *T. cruzi* DTUs based on five molecular markers. ^1^ Souto *et al*., 1996, ^2^ Liarte *et al*., 2009, ^3^ Brisse *et al*., 2000, ^4^ Duffy *et al*., 2009 and ^5^ Falla *et al*., 2009.

Molecular detection was performed amplifying the variable region of kinetoplast DNA (kDNA) using primers 121 (5′AAATAATGTACGGGKGAGATGCATGA3′) and 122 (5′GGTTCGATTGGGGTTGGTGTAATATA 3′), and tandem repeat satellite region from *T. cruzi* using primers cruzi1 (5′ASTCGGCTGATCGTTTTCGA3′) and cruzi2 (5′AATTCCTCCAAGCAGCGGATA 3′). Molecular characterisation of *T. cruzi* was carried out using five molecular markers that have been previously evaluated. The intergenic region of the non-transcribed mini-exon gene using primers TCC (5′CCC CCC TCC CAG GCC ACA CTG 3′), TC1 (5′GTG TCC GCC ACC TCC TTCGGG CC 3′) and TC2 (5′CCT GCA GGC ACA CGT GTG TGT G 3′); all mini-exon gene PCR assays were performed using two primers instead of a multiplex PCR assay to determine the presence of mixed infections. Thus, PCR was employed using TCC-TC1 and TCC-TC2 primers. The variable domain D7 of the rDNA 24Sα subunit using primers D71 (5′AAG GTG CGT CGA CAG TGT GG 3′) and D72 (5′ TTT TCA GAA TGG CCG AAC AGT 3′), the primers V1 (5′CAA GCG GCT GGG TGG TTA TTC CA 3′) and V2 (5′TTG AGG GAA GGC ATG ACA CAT GT 3′) amplifying the 18S rRNA ribosomal sequence of *T. cruzi* and the repetitive region of satellite DNA using primers Diaz8 (5′ TGT TCA CAC ACT GGA CAC CAA 3′) and TcSat4 (5′GCA GCC GCT CGA AAA CTA TCC 3′) by conventional PCR and qPCR using primers TcZ1 (5-′CGAGCTCTTGCCCACACGGGTGCT3)′ and SatRv (5′TTCAGRGTTGTTTGGTGTCCAGTG3′) from *T. cruzi* (Figure 1). For all molecular markers, the amplification reactions were performed in a total volume of 21 µL. This reaction consisted of 1X of Taq polymerase amplification buffer (100 mM Tris-HCl, pH 8.3; Invitrogen), 100 mM dNTPs solution, 50 mM MgCl_2_ solution, 5 Units/µL of Taq polymerase platinum (Invitrogen), 50 pM of each primer, 3 µL of DNA template and water to give a final total volume of 21 µL. Amplification cycles were applied in an automatic thermocycler (BIORAD iCycler) as previously reported [24], [25], [26]. The real-time PCR assay was performed using 2X Supermix SYBR green iQ (100 mM KCl, 40 mM Tris-HCI, pH 8.4, 0.4 mM dNTP, 50 U/mL of iTaq polymerase, 6 mM MgCl2, SYBR Green I, 20 nM fluorescein); 50 pM of TcZ1 and SatRv primers and 3 µL of DNA, the thermal profile and acquisicion of fluorescence was used as previously reported [27]. Molecular identification of genotypes in TcI was accomplished using primers 1-A (5′TGT GTG TGT ATG TAT GTG TGT GTG 3′), 1-B (5′ CGG AGC GGT GTG TGC AG 3′) for the identification of genotype Ia with an amplification product of 288 bp; 2-A (5′TGT GTG TGT GTA TGT ATG TAT GCT 3′), 2-B (5′GGA ACA CAC GCG ACT AAT-3′) for the identification of genotype Ib with an amplification product of 250 bp; 4-A (5′CTG CAG GCA CAC GTG TGT 3′), 4-B (5′AAA AGA CGG GAA AAA AGC AA 3′) for the identification of genotype Id with an amplification product of 200 bp (Figure 1). The amplification reactions were performed in a total volume of 20 µL. This reaction consisted of 1X of Taq polymerase amplification buffer (100 mM Tris-HCl, pH 8.3; Invitrogen), 100 mM dNTPs solution, 25 mM MgCl_2_ solution, 5 Units/µL of Taq polymerase platinum (Invitrogen), 10 µM of each primer, 3 µL of DNA template and water to give a final total volume of 20 µL. Amplification conditions were independently established as previously reported [12]. Each reaction was carried out in duplicate, and twenty microliters of PCR product for each reaction were analysed by electrophoresis on a 2% agarose gel and visualised by staining with ethidium bromide. Positive controls were always included in the PCR assays using CG strain (TcI), VS strain (TcII), CM17 strain (TcIII), CANIII strain (TcIV), MN cl 2 (TcV), CL Brener strain (TcVI) and 444 (*T. rangeli* strain).

### Characterisation of presence/absence of electrocardiographic, radiologic and echocardiogram alterations

Seventeen cardiac abnormalities were evaluated during electrocardiographic, radiologic and echocardiographic characterisation of each patient. The 17 alterations are as follows: right bundle-branch block (1), left bundle-branch block (2), left anterior fascicular block (3), left posterior fascicular block (4), ventricular premature beats (5), first degree atrioventricular block (6), Mobitz type I atrioventricular block (7), sinus bradycardia (8), primary ST-T changes (9), abnormal Q-waves (10), low voltage QRS (11), atrial fibrillation (12), Mobitz type II atrioventricular block (13), complete atrioventricular block (14), complex ventricular arrhythmias (15), evidence of regional wall motion abnormality (16), reduced global left ventricular function and increased cardiothoracic ratio (17) [22]. Variables were taken as categorical and the result was analysed by presence or absence of each abnormality. The prevalence of the abnormalities was determined based on the 240 patients evaluated. Independence tests using Chi-square test (p<0.05) were performed in 20 random TcI and 20 TcII patients to find possible associations between the presence/absence of cardiac abnormalities in patients characterised as TcI, TcII-TcIV and possible mixed infections TcI/TcII-TcIV; also to evince statistically significant differences in the effect of specific DTUs with the presence/absence of cardiac abnormalities. Student t test (p<0.05) was developed followed by a Tukey test (p<0.05) to observe the statistically mean differences among each cardiac abnormality and *T. cruzi* DTUs. Lastly, to ensure that all the results obtained were not attributed to randomness, all the results were randomized in PopTools 3.1.0 with 10,000 replicates (p<0.05).

## Results

### *T. cruzi* molecular detection and characterisation

**Figure 2:**
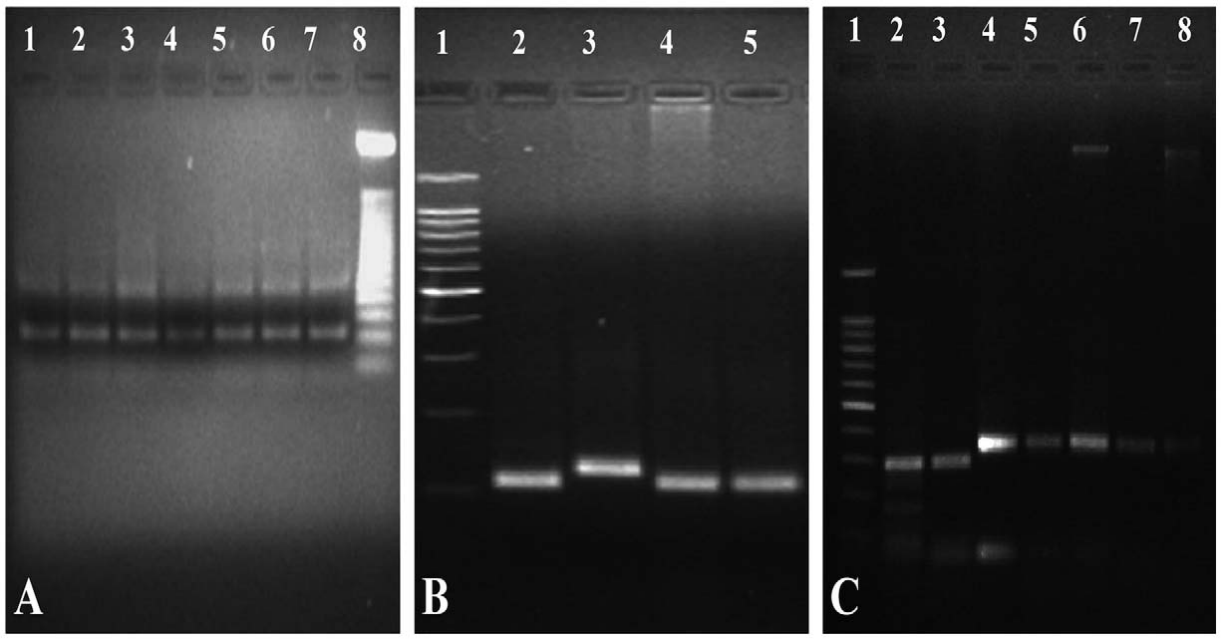
Amplification products of the DNAst, intergenic region of mini-exon gene and 24Sα rDNA region performed. Figure 2A. Amplification product in 2% agarose gels of DNAst. Lane 1-6 Patients 111- 116 TcII-TcVI, Lane 7. Patient 87, Lane 8. 50-bp Weight Marker. Figure 2B. Amplification product in 2% agarose gels of the D7 24Sα rDNA region. Lane 9. 100-bp Weight Marker, Lane 10. Patient 87 TcI, Lane 11. Patient 98 TcII-TcVI, Lane 12–13. Patients 101-107 TcI. Figure 2C. Amplification product in 2% agarose gels of the intergenic región of mini-exon gene. Lane 1. 100-bp Weight Marker, Lane 2. Patient 12 TcII-TcVI, Lane 3. Patient 34 TcII-TcVI, Lane 4–8. Patients 33-34-35-36 TcI.

Out of 240 samples, 168 (70%) were positive by kDNA amplification for *T. cruzi* DNA detection. When satellite DNA PCR for typing *T. cruzi* DTUs was performed 177/240 (74%) samples showed positive amplification, with 131/177 (74%) patients being classified as TcI and 46/177 (26%) as TcII-TcVI (Figure 2A). When the intergenic region of mini-exon gene was performed, 132/240 (55%) samples were positive, 95/132 (72%) samples corresponding to TcI and 21/132 (16%) corresponding to TcII-TcVI; also, 16/132 (12%) samples showed both patterns of amplifications and were considered mixed infections TcI/TcII-TcVI. (Figure 2C). In regard to the variable domain D7 rDNA 24Sα subunit, positive amplification was observed in 161/240 samples and 136/161(71%) showed positive amplification for TcI, 25/161 (15%) showed positive amplification for TcII-TcVI. (Figure 2B). Regarding the qPCR strategy to genotype *T. cruzi*, this assay was only performed to confirm TcIII and TcIV according to the melting temperatures previously established [25].

### *T. cruzi* I genotypes and TcII-TcVI DTUs identification

In the single infections, TcI genotypes were detected (95/240), and amplification results were observed for TcIa (46/95) and TcId (8/95) genotypes. In the mixed infections, only genotype Id was detected (16/22). Due to the low sensitivity of this molecular marker no amplification was observed in some samples that were positive by amplification of the intergenic region of the mini-exon gene.

**Figure 3:**
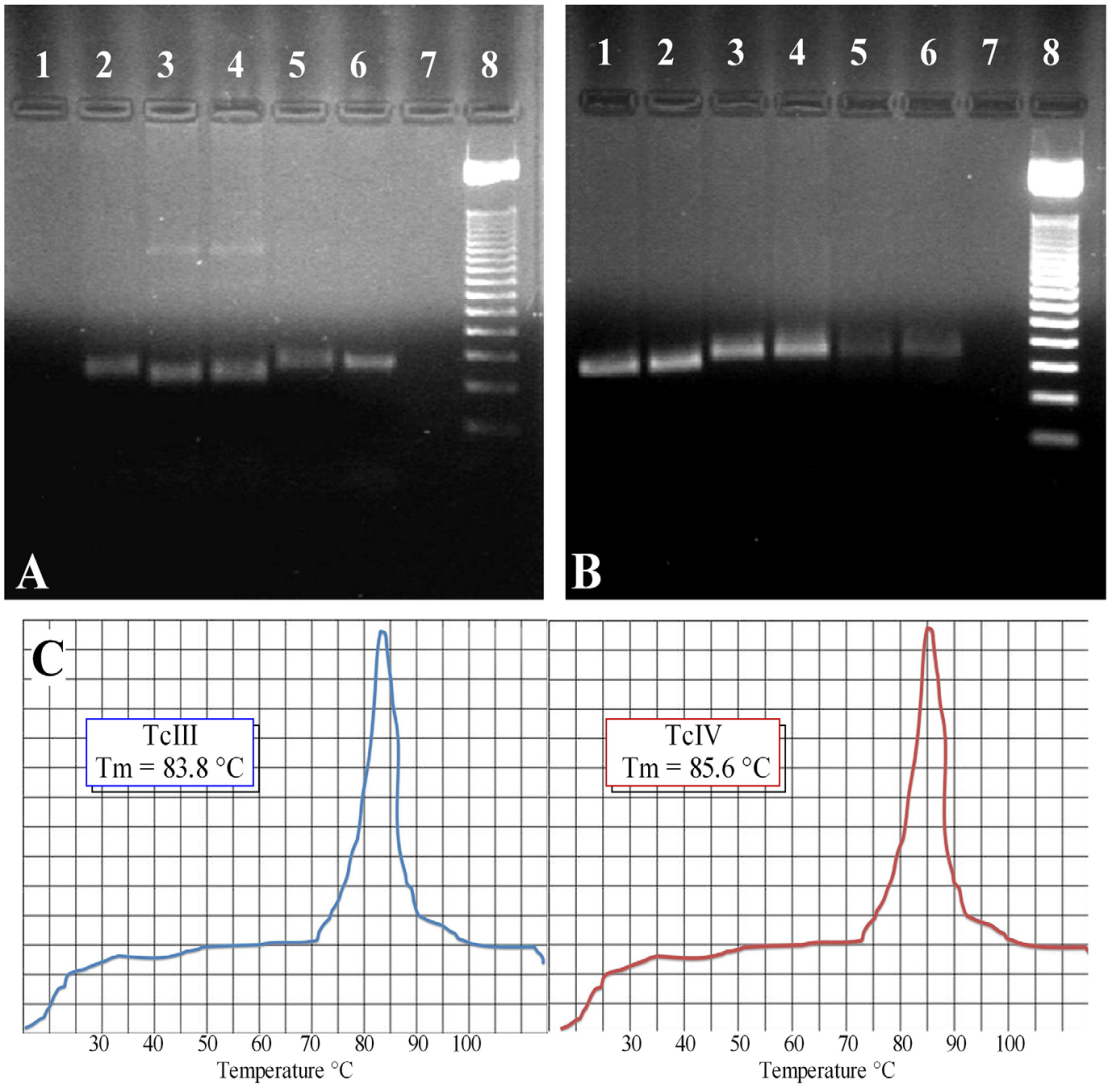
Amplification products of the 18S, 24Sα rDNA and qPCR DNAst Tm peaks for the detection of TcIII and TcIV DTUs. Figure 3A. Amplification products in 2% agarose gels of D7 24Sα rDNA region in *T. cruzi*. Lane 1. Negative control, Lane 2. Patient 54 TcIV, Lane 3. Patient 76 TcIII, Lane 4. Patient 112 TcIII, Lane 5. Patient 212 TcII, Lane 6. Patient 12 TcII, Lane 7. Negative control, Lane 8. 50-bp Weight Marker. Figure 3B. Amplification products in 2% agarose gels of 18S rDNA region in *T. cruzi*. Lane 1. Patient 57 TcIV, Lane 2. Patient 54 TcIV, Lane 3. Patient 76 TcIII, Lane 4. Patient 112 TcIII, Lane 5. Patient 212 TcII, Lane 6. Patient 12 TcII, Lane 7. Negative control, Lane 8. 50-bp Weight Marker. Figure 3C. Melting temperature peaks of TcIII and TcIV samples by qPCR real-time.

When the rDNA 18S region and 24Sα D7 domain patterns of amplification were obtained 24/25 patients were infected with TcII in the single infections. Regarding the results of mixed infections obtained by Mini-exon TcI/TcII-TcVI a markedly low frequency of TcII was observed, only 1/22 patients were infected with TcII, 5/22 with TcIII and 10/22 with TcIV, all in mixed infection with genotype TcId (Figure 3A–3B). The presence of TcIII and TcIV was corroborated using the melting temperature analysis in the qPCR assays based on the satellite DNA region showing that those samples characterized as TcIII (5/22) and TcIV (10/22) were ascertained those DTUs (Figure 1; Figure 3C).

### Characterisation of cardiomyopathy and correlation with molecular characterisation of the *T. cruzi* infection

**Figure 4:**
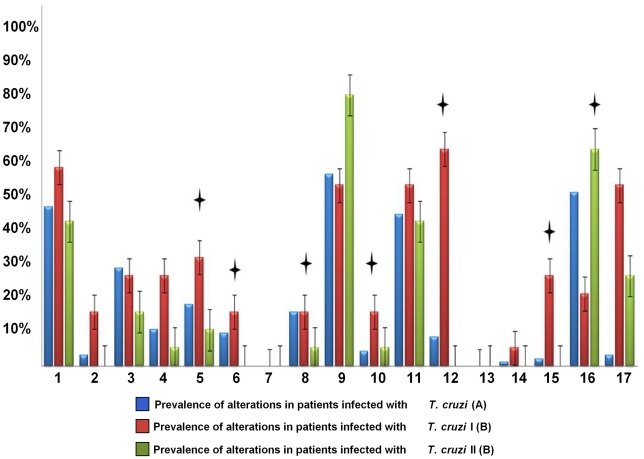
Prevalence of electrocardiographic, radiologic and echocardiogram alterations in chronic Chagasic patients from Colombia. (A) Prevalence of alterations based on 240 chronic Chagasic patients, (B) Prevalence of alterations based on 20 selected chronic Chagasic patients. Right bundle-branch block (1), left bundle-branch block (2), left anterior fascicular block (3), left posterior fascicular block (4), ventricular premature beats (5), first degree atrioventricular block (6), Mobitz type I atrioventricular block (7), sinus bradycardia (8), primary ST-T changes (9), abnormal Q-waves (10), low voltage QRS (11), atrial fibrillation (12), Mobitz type II atrioventricular block (13), complete atrioventricular block (14), complex ventricular arrhythmias (15), evidence of regional wall motion abnormality (16), reduced global left ventricular function and increased cardiothoracic ratio (17). (*) Statistically significant comparisons of TcI vs. TcII cardiac alteration using Tukey test.

Statistical analyses were performed in terms of trying to define the correlation between *T. cruzi* genetic variability and the presence/absence of cardiac abnormalities in chronic Chagasic patients. The prevalence of the cardiac alterations was estimated (Figure 4). Due to the predominance of patients infected with TcI and the low number of patients infected with TcII, twenty random TcI and 20 TcII samples were selected to conduct the statistical analyses. Independence test (Chi-square p<0.05) showed associations between the presence/absence of cardiac alterations and the infection by specific *T. cruzi* DTUs (p = 0.037 for TcI and p = 0.039 for TcII) Student t-tests showed that there were mean differences in the presence of cardiac alterations in patients characterised as TcI or TcII (p = 0.033). The prevalence of cardiac alterations was estimated in TcI and TcII based on the 20 samples previously selected (Figure 4). Significant and non-significant mean pair-wise comparison using Tukey test was developed on the 20 samples selected showing that the prevalence of most cardiac alterations was elevated depending on TcI or TcII infection (Figure 4). Likewise, to ensure that the results were not attributed to randomness, all the data was randomised in PopTools 3.1.0 with 10,000 replicates and it was observed that the data were not attributed to randomness (p = 0.037). The cardiac abnormalities were also assessed comparing the prevalence of these alterations between the genotype TcIa and the genotype TcId, Chi-square test based on 8 random TcIa samples and 8 TcId samples were performed showing association between cardiac alteration and TcIa (p = 0.011) and no association between cardiac alteration and TcId (p = 0.061). Furthermore, the t-student test showed strong mean differences between cardiac alterations of TcIa and TcId (p = 0.023) demonstrating that the genotype related to the domestic cycle of transmission presents more cardiac alterations than those patients infected with the genotype related to the sylvatic cycle of transmission.

## Discussion

The main purpose of defining *T. cruzi* nomenclature must be related with the biological, clinical and pathological characteristics associated with specific populations of *T. cruzi* [14], [18]. So far to our knowledge, few correlations reported have been evidenced in the difference of the host humoral response to specific *T. cruzi* genotypes; however, these findings were flawed because of the low reliability of the diagnostic tests used, leading to a high proportion of false negatives due to variability in the *T. cruzi* strain used for the diagnosis, the incrimination of TcI with severe forms of myocarditis in cardiac samples from chronic chagasic patients in Argentina and no specific clinical manifestation related to *T. cruzi* DTUs in Bolivian chagasic patients show the pleomorphism of *T. cruzi* [23], [28], [29], [30]. Regarding the genetic variability of the parasite, prognosis markers based on mitochondrial genes where the presence of specific mutations can trigger the complications of the chronic phase of disease in asymptomatic patients have also been demonstrated [31], [32]. Despite the genetic variability, it is important to consider the presence of *T. cruzi* clones that can be found in different tissues. Several studies have demonstrated a specific histiotropism of *T. cruzi* in mice showing differences in the pathological, immunological and clinical features the parasite can elicit in the host [18], [33], [34]. Moreover, some authors have shown that the *T. cruzi* population in a patient's bloodstream could be dissimilar to the parasite population that causes tissue damage [35]. Differences were found in *T. cruzi* populations in the bloodstreams of patients with chronic Chagasic cardiomyopathy and of Chagasic patients without cardiomyopathy [36]. Also, microsatellite analyses have shown multiclonality in samples of heart and in the bloodstream of infected patients [37], [38] demonstrating that probably specific populations of *T. cruzi* can determine the disease outcome.

The presence of TcIII and TcIV could possibly be explained by the selection of *T. cruzi* in the amplification procedures; as mentioned before the predominant DTU in Colombia is TcI and the amplification procedures are being subjected to this specific DTU not allowing the amplification in the axenic culture of other low parasite density DTUs. This has been evidenced in *T. cruzi* isolates that are considered mixed infections especially in congenital cases [37], [39], [40], [41]. Another factor is that sylvatic reservoirs might be selecting specific clones of *T. cruzi*. Recently, the possible association between *T. cruzi* DTUs and sylvatic reservoirs has been shown. TcI is related to opossums in the arboreal ecotope and TcII-TcVI to armadillos in the terrestrial ecotope where it was not possible to find opossums infected with TcII-TcVI, suggesting the possible selection of *T. cruzi* DTUs in the reservoirs [38], [39], [40]. Selection of *T. cruzi* populations could also be caused by the factorial contact of reduviid insects in their bloodmeal with humans and sylvatic reservoirs, such that they can acquire different *T. cruzi* populations each time they feed [14], [16].

Most of the patients recruited in this study came from an endemic region of Colombia (Santander) where wild reservoirs and sylvatic triatomines have been reported [19], [20], [42]. This diversification of sylvatic triatomines could explain the unexpected transmission of the foreseen genotypes TcIII and TcIV. Also, the possible interaction of sylvatic triatomines in the domestic cycle of *T. cruzi* transmission might be an explanation for the appearance of TcII, TcIII and TcIV in the chronic Chagasic patients. Infection of TcII-TcIV in *Rhodnius prolixus* and *Panstrongylus geniculatus* has been reported and might explain the presence of TcII-TcIV and its association with the domestic cycle where parasites with these DTUs are infecting the patients [43]. Some hypothesis show that reservoirs from the arboreal ecotopes such as didelphids and primates are always infected with TcI and those associated with the terrestrial ecotopes such as armadillos that have been found infected with TcIII and some sylvatic rodents are infected with TcII-TcVI [44]. Recent reports show that this distribution is not absolute because *Monodelphis brevicaudata* and *Philander frenata* have been found infected with TcIII and *D. aurita*, primates and wild non-human primates have been infected with TcII, TcI and TcIV respectively [45], [46], [47], [48]. The reservoirs play an important role in the epidemiology of Chagas disease and represent the basis of finding patients infected with TcIII and TcIV showing rodents such as *P. semispinosus* and *Rattus rattus* infected with TcIII or TcIV in Colombia [49], [50].

The most interesting observation is the presence of TcIII (5/22 patients) and TcIV (10/22 patients) in the chronic Chagasic Colombian patients. Hybridisation events have been observed in *T. cruzi* based on the use of satellite DNA, rRNA sequences and phylogenetic inferences [5], [51], [52], [53], [54]. TcIII and TcIV are considered the Zymodeme III related to the sylvatic cycle of transmission in the Amazon basin and also related to TcI [55], [56], [57]. TcIII and TcIV are reported as a possible product of an event of recombination between TcII and TcI. Herein, we found the presence of genotype Id in mixed infections and the decrease of the number of patients infected with TcII. We recently discovered the existence of genotypes within TcI isolates [8], [9], [12]. These results have been corroborated using the internal transcribed spacer 2 (ITS-2) where three genotypes were clearly grouped [13]. A set of 48 loci microsatellite analyses corroborated the sylvatic and domestic-peridomestic genotypes (Ia and Id) and the recent report of the genotype TcIe supports the idea that TcI is a really diverse DTU that requires further investigation in order to obtain hidden information of this DTU [7], [9]. Our results demonstrate the presence of TcIV in infected patients as previously reported in Venezuela [58]. In addition, we report the presence of TcIV in human mixed infection with genotype Id. Recent studies have shown the presence of TcIa and TcId genotypes in chronic chagasic patients from Argentina. Interestingly, the most prevalent genotype is Ia in bloodstream and Id more prevalent in cardiac tissue explants suggesting TcI genotype histiotropism [29]. Our results agree with these findings when TcIa was found in 46 patients and TcId in 24 patients (8 from single infections plus 16 from mixed infections).

Statistical significance was obtained when independence tests were performed using categorical data for the presence or absence of cardiac alterations detected by the electrocardiographic, radiologic and echocardiograpic methods. Independence was observed when TcI and TcII were determined reflecting that the genetic variability in *T. cruzi* may represent an important factor for disease installation. Moreover, the findings of this study demonstrate that TcI is the predominant genotype associated with manifestations of cardiomyopathy in chronic Chagasic patients. These results have already been confirmed where severe myocarditis was found in patients from Argentina infected with TcI and moderate myocarditis was caused by TcV and TcVI despite of TcII can also cause a lower grade of severe myocarditis [29]. The *T. cruzi* population distribution in the bloodstream and in the cardiac tissue has been shown to be quite different in previous studies [18], [33], [34]. Therefore, it is now really necessary to conduct studies based on the use of cardiac tissue and bloodstream samples to compare the *T. cruzi* genotypes that are circulating in Colombian Chagasic patients and those that are probably involved in producing organ damage in infected patients.

In conclusion, we report for the first time the presence of TcIII and TcIV in chronic Colombian Chagasic patients. We also confirm the presence of TcI and TcII in chronic Chagasic patients, and found that TcI is associated with more cardiomyopathy abnormalities than TcII. In addition, we describe the predominance of TcI in Colombia and the mixed infection in the same patient with TcI/TcII-TcIV. It is important to consider that our study was accomplished in a restricted area and the whole *T. cruzi* diversity in chronic Chagasic patients was not considered, also the detected genotypes in bloodstream and populations that cause organ failure may be dissimilar as has been previously reported in Colombia: TcI in bloodstream and TcII in cardiac tissue in a same patient [20], [29], [33], [34]. New studies regarding most of the endemic areas from Colombia and molecular characterization directly from infected organs are required to determine the *T. cruzi* populations circulating in Colombian patients. New studies are also necessary to understand the specific *T. cruzi* populations that are generating the tissue damage in the infected patients.
